# Will Multidisciplinary Collaboration Reduce the Disability Rate of Diabetic Foot (2009–2019)?—A Study Based on the Perspective of Organizational Reform

**DOI:** 10.3389/fpubh.2021.760440

**Published:** 2021-10-08

**Authors:** Mengchi Hou, Xue Gong, Wenhu Chang, Jie Dong, Feifei Zhao, Zhili Ji, Rui Guo

**Affiliations:** ^1^School of Public Health, Capital Medical University, Beijing, China; ^2^Beijing Luhe Hospital, Capital Medical University, Beijing, China; ^3^Beijing Anzhen Hospital, Capital Medical University, Beijing, China

**Keywords:** multidisciplinary, disability rate, diabetic foot, organizational reform, assess

## Abstract

**Objectives:** Discuss the experience and practice of multidisciplinary cooperation of diabetic foot in China and analyze its impact on the quality of care.

**Methods:** This study observed the medical procedure by interviewing 12 key personnel in-depth. We extracted data from medical records and assessed the effect of MDT in three dimensions: quality, efficiency, and cost, to eventually achieve a final conclusion.

**Results:** The studied reform includes the following three aspects: the adjustment of hospital buildings layout and disciplines, one-stop outpatient, and one-stop inpatient service. After the multidisciplinary collaboration, the rate of above-knee amputation is reduced by 3.63%, the disability score per 100 diabetic foot patients decreases by 6.12, the average length of stay decreases significantly, and the cost of hospitalization shows an increasing trend.

**Conclusions:** Multidisciplinary collaboration is performed based on spatial layout adjustment and clinical pathway optimization, which provide more comprehensive and integrated care than a general medical team or a single specialist, thereby reducing the rate of disability, shortening the length of hospitalization. Besides, the new measurable indicator called disability score per 100 diabetic foot patients has been verified to evaluate the living ability of patients after surgery. This paper provides a reference for organizational reform of multidisciplinary diseases to support treatment and management of other multiorgan diseases.

## Introduction

The International Working Group on the Diabetic Foot (1) defined diabetic foot in 2020 as “Infection, ulceration, or destruction of tissues of the foot of a person with currently or previously diagnosed diabetes mellitus, usually accompanied by neuropathy and/or peripheral artery disease in the lower extremity.” Resulting in massive economic consequences to patients, families, and society (2), several factors have led diabetic foot to the most severe complication of diabetes mellitus worldwide. Firstly, the prevalence of diabetic foot remains at a high level. IDF Diabetes Atlas 9th edition (2019) mentioned that the global prevalence of diabetic foot varies between 3% in Oceania to 13% in North America, with a global average of 6.4% (3). Secondly, with a poor prognosis, the diabetic foot has been one of the main causes of disability and death in diabetic patients, which overwhelmed the mortality and disability rate of most common cancers (except for lung cancer, pancreatic cancer, etc.) (4). It is estimated that every 20 s somewhere in the world, someone loses a leg due to the complications of diabetes (5). The annual mortality rate of diabetic foot ulcer patients is as high as 11%; meanwhile the mortality rate of amputation patients is as high as 22% (4). Thirdly, the medical cost of the diabetic foot could be a huge burden. Diabetic patients who suffer foot ulcers bear health expenditures five times higher than those ulcer-free patients (3). In low-income countries, the cost of complex diabetic foot ulcer treatment is equivalent to the patient's 5.7 years of income, pushing patients and their families into predictable bankruptcy (6).

Traditional single-specialized treatment can hardly achieve the clinical goals of diabetic foot treatment, considering that the diagnosis and treatment of diabetic foot involve endocrinology, vascular surgery, orthopedics, burn surgery, infection and general surgery, etc. Effective efforts to prevent and treat diabetic foot disease requires a well-organized team applying a holistic approach, which regards ulcer as a sign of multiorgan disease, and will surely involve various disciplines (5). The multidisciplinary team (MDT) includes clinicians with different roles, specialization, and expertise, which allows creating a network with the patient at the center of the decision process, and the final aim of this path was to make a correct diagnosis and provide patients with the best possible treatment (7). Although debated, the rate of amputation has been considered an indicator of the quality of diabetic foot care (8). Studies around the world also show that MDT can effectively reduce the rate of amputation and improve the quality of life of patients. For example, in rural England, an MDT led by vascular surgeons was established, and the rate of lower limb amputations was consequently reduced from 412/100,000 to 15–44/100,000 (8). The establishment of MDT has also resulted in a decrease in hospitalization due to diabetic foot (9). It has been suggested that it can even be cost-effective to introduce an MDT (10). However, the results of studies on the actual utility of MDT have not been uniformly positive. Some researchers have also found that MDT may not actually have a positive effect on the clinical efficacy of patients.

The achievement of a decreased amputation rate in patients with diabetes has been associated not only with the setup of an MDT approach but also with other factors, including improvements in organizational structures and healthcare processes and the implementation of the clinical pathway (11). In China, Beijing Luhe Hospital started an organizational reform in 2015. To improve organizational structure, Luhe Hospital has reconstructed traditional departments and built up an organ-system-based, disease-coring multidisciplinary health care project. They redesigned standardized and comprehensive clinical pathways of certain multidisciplinary diseases to break through the barriers between traditional disciplines (especially internal medicine and surgery). The spatial design of inpatient and outpatient departments have also been rearranged for the purpose of a patient-centered and integrated service procedure. As one of the earliest diseases involving the reformation in Luhe Hospital, the diabetic foot is selected to study the Chinese multidisciplinary cooperation mode and analyze its effects in efficiency, quality, and cost in clinical practice.

## Methods

### Data Sources

An observational method was used to get the medical procedures with MDT. Meanwhile, the president and 11 clinical department directors participated in in-depth interviews about the reform, four of which were from endocrinology and vascular surgery. Data about the diabetic foot was extracted from the medical records of Luhe Hospital. Firstly, the search strategy was clearly defined. There were two strategies: (1) The main diagnosis was “diabetic foot”; Or (2) The main diagnosis was “diabetes” or “foot gangrene/ulcer,” and other diagnoses were “diabetic foot.” Secondly, data were extracted with the assistance of Luhe Hospital's professional staff in the department of medical records, and data was from January 1, 2009, to December 31, 2019. Finally, the two researchers checked and cleaned up data to ensure quality. There were a total of 762 patients with diabetic foot included in this study.

### Data Analysis

Wagner classification, which is mostly used to grade the severity of patients with diabetic foot (12), is used to divide the hospitalized patients into three groups: mild, moderate, and severe (Table 1). Besides, based on whether surgery was performed, patients are also divided into two categories: amputated and non-amputated patients. To better analyze the effect of MDT, we classify the operations that affect the life function of diabetic foot patients into three categories: phalange/metatarsal resection, below-knee amputation, and above-knee amputation (Table 2).

**Table 1:** Wagner ulcer classification system.

**Grade**	**Lesion**	**Group**
0	No open lesions; may have deformity or cellulitis	Mild
1	Superficial diabetic ulcer (partial or full thickness)	
2	Ulcer extension to the ligament, tendon, joint capsule, or deep fascia without abscess or osteomyelitis	Moderate
3	Deep ulcer with abscess, osteomyelitis, or joint sepsis	
4	Gangrene localized to the portion of the forefoot or heel	Severe
5	Extensive gangrenous involvement of the entire foot	

**Table 2:** Categories.

**Category**	**Method**
Phalange/metatarsal resection	such as phalange amputation, phalange disarticulation, multi-phalange amputation, metatarsal lesionectomy, disarticulation of metatarsal-phalangeal joint, metatarsal sequestrectomy, phalange sequestrectomy, phalange wedge osteotomy, interphalangeal amputation, pedis sequestrectomy
Below-knee amputation	such as foot disarticulation, ankle joint disarticulation, leg amputation through tibia and fibula, leg disarticulation through tibia and fibula
Above-knee amputation	such as leg disarticulation, knee joint disarticulation, thigh amputation, thigh disarticulation

We describe sociodemographic characteristics of patients with diabetic foot, including gender, age, diabetes history, groups by severity, and glycosylated hemoglobin. We individually assess the differences in treatment before and after reform in mild, moderate, and severe groups. We analyze the effect of MDT on quality, efficiency, and cost. Quality indicators include (1) phalange/limb amputation rate; (2) disability score per 100 diabetic foot patients. Efficiency indicators include (1) average length of stay; (2) mean operation time; (3) average length of post-operative stay. Cost indicators include (1) average hospitalization cost. We use the Statistical Package for the Social Sciences (SPSS) version 26.0 to do the statistical analysis. We perform χ^2^ test or *t-*test and choose *P* < 0.05 as the significant statistical thresholds (2-tailed).

## Results

### Organizational Reform

The successful implementation of organizational reform requires both top management commitment as well as a bottom-up approach that ultimately places decision making at the place in the organization where the work is performed (13). Luhe Hospital followed this point of view to carry out organizational changes. And the organizational reform implemented by Luhe Hospital was different from MDT. Clinical department director A (endocrinology) stressed: “*The model of Luhe Hospital is an elaborative design based on the MDT. It is not only the collaboration of experts but also a comprehensive adjustment from space to working methods, that is, the reconstruction of space structure and processes. For example, ultrasonography enters each ‘Clinic District,' instead of sharing the same ultrasound examination room for all outpatient departments as before.”* Clinical department director B (vascular surgery) emphasized: “*Luhe' organizational reform is similar to MDT in principle but different in nature. MDT was more like a situation where one person was in trouble, and everyone helped to support them. The layout was relatively random. Luhe's organizational reform takes disease as the core. For example, endocrinology and vascular surgery are jointly responsible for diabetic foot, which is a win-win cooperation. Figuratively speaking, in the organizational reform, endocrinology and vascular surgery are husband and wife, living together. While in MDT, they were neighbors.”*

Its specific reform includes the following three aspects: First, the adjustment of hospital buildings layout and disciplines. Luhe Hospital adopted the “Clinic District” model with patient-centered to allow patients to complete the whole process of medical treatment in the same area. The original 40 clinical departments were integrated into 28 clinical centers. For instance, the original endocrinology, vascular surgery, rheumatology and immunology, and diabetes research institute had been integrated into the “Center of Endocrine, Metabolism and Immune Disease,” which concentrates on the diabetic foot and other diseases. And the related departments of the center were distributed on the same floor to avoid patients wasting time between different buildings or floors within the hospital.

**Figure 1:**
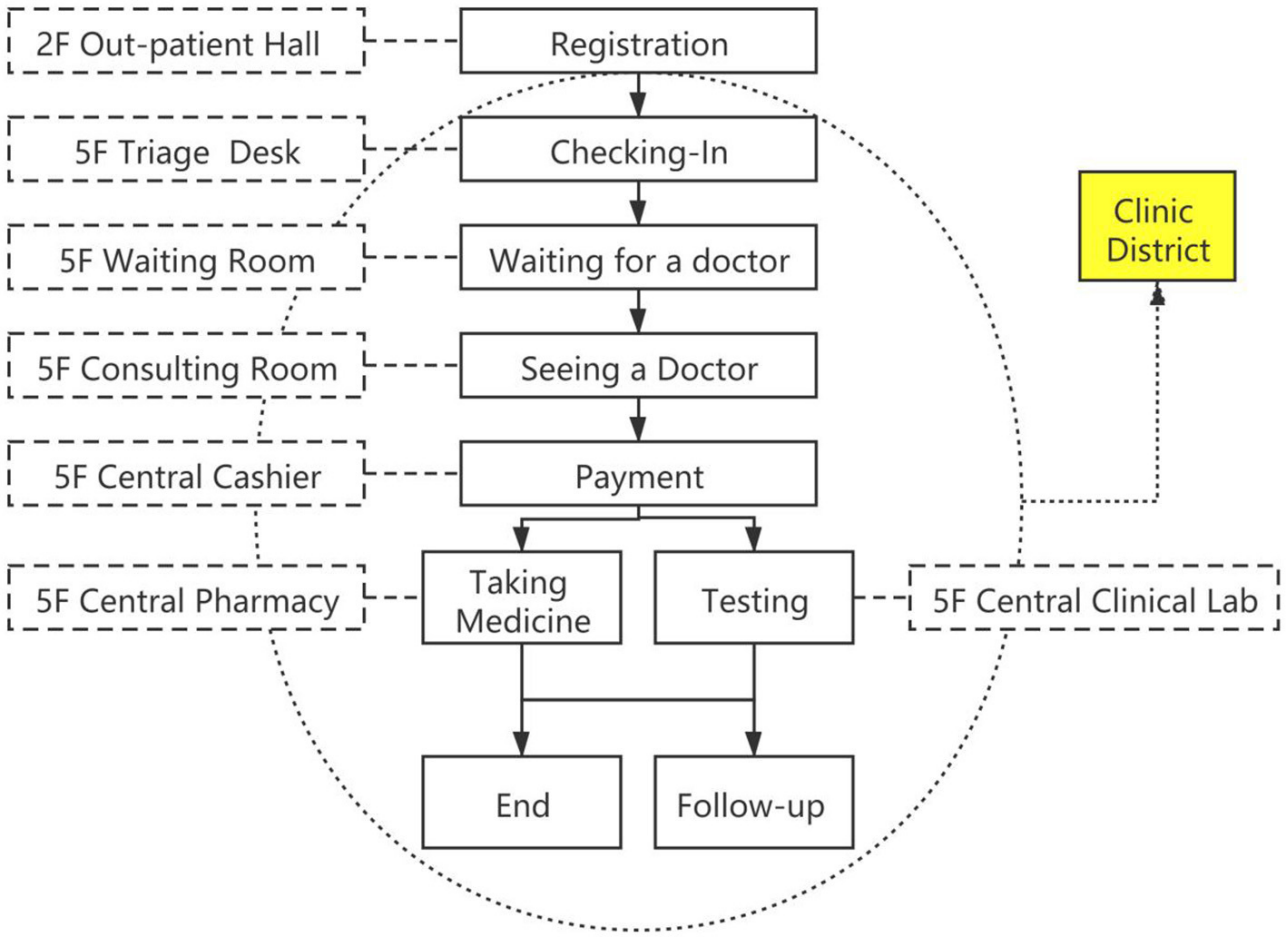
“One-station” outpatient service.

Second, one-stop outpatient service (as Figure 1 shows). The outpatient departments were divided by the organ system, and the physician and the surgeon made a joint visit to evaluate the patient's condition and formulate the therapeutic schedule. In addition, each center had set up the necessary clinical laboratory department, and patients can complete most of the examination, diagnosis and treatment procedures in the “Clinic District.” The center offers a streamlined service and truly implements the concept of “patient-centered”.

**Figure 2:**
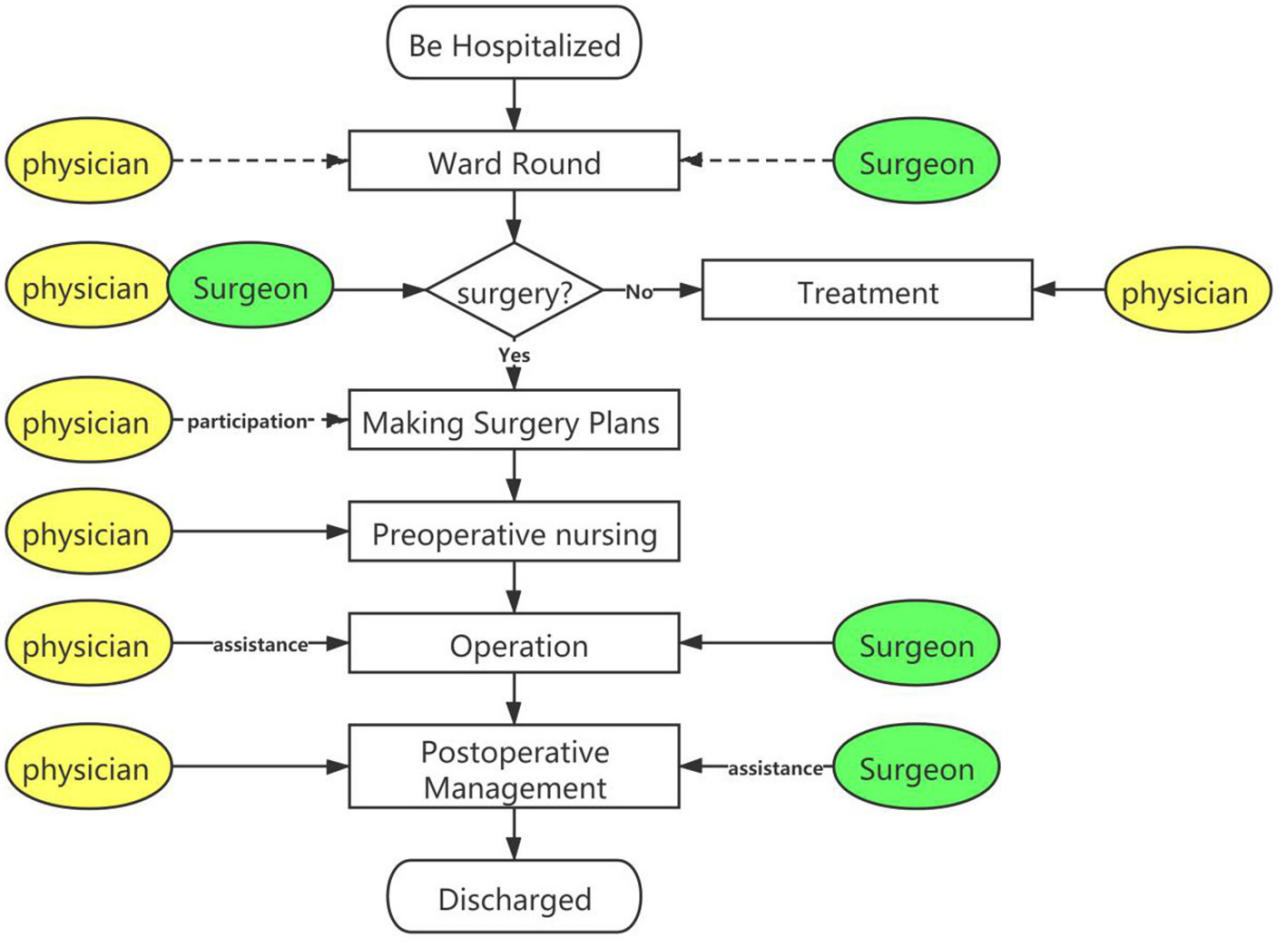
“One-station” inpatient service.

Third, one-stop inpatient service (as Figure 2 shows). Traditionally, wards in different departments were managed completely separately. In the Luhe hospital, the boundaries between different wards were blurred, and the “one bed” system was implemented inside the center. That is to say, hospitalized patients can be admitted to whichever ward. Multidisciplinary physicians make a joint ward round, shift work, and co-management hospitalized patients. In joint surgery, physicians locate the lesion, surgeons take charge of the operation, and post-operative care was co-managed by multidisciplinary physicians. Before organizational reform, each department worked separately. Patients revolved around doctors and received separate medical services. While after it, the departments work jointly in the “Clinic District,” doctors revolve around patients and provide continuous medical care.

The reform makes sense for patients with diabetic foot. In the outpatient department, an endocrinologist and a vascular surgeon are responsible for the whole process management of diabetic foot patients. They make the joint visit and invite doctors from cardiology, neurology, nutrition, nephrology, and other disciplines to make a joint decision when necessary. Multidisciplinary physicians also make ward rounds together at regular intervals in the inpatient department. Specific contexts are as follows. (1) Cardiology: evaluate the cardiovascular system, monitor and treat the blood pressure timely to avoid diabetic cardiovascular diseases. (2) Neurology: evaluate the limb function, detect and treat the peripheral neuropathy timely to reduce the nerve damage of diabetic foot patients. (3) Nutrition: evaluate the nutrition of patients and modify the diet. (4) Nephrology: evaluate the renal function, detect and treat diabetic nephropathy timely.

With the deepening of organizational reform, the process of diagnosis and treatment has been significantly improved, the cooperation between physicians and surgeons has become more tacit, and it has become more convenient for patients to seek medical treatment, achieving a win-win situation for doctors and patients.

### Baseline Data

From 2009 to 2019, there was a total of 762 patients discharged from the hospital with diabetic foot, including 184 patients from 2009 to 2014 and 578 patients from 2015 to 2019. Table 3 shows the sociodemographic characteristics of diabetic patients, including gender, age, diabetes history, groups by severity, and glycosylated hemoglobin. Results show there is no statistical significance, except for severity (*p* = 0.017) and age (*p* < 0.001).

**Table 3:** Sociodemographic characteristics of diabetic patients.

	**2009–2014**	**2015–2019**	***t* (*χ^2^*)**	***P-*value**
Gender (*n* = 762), *n* (%)			(0.877)	0.349
Male	102 (55.4)	343 (59.3)		
Female	82 (44.6)	235 (40.7)		
Diabetes (*n* = 677), *n* (%)			(0.105)	0.745
Yes	96 (97.0)	553 (95.7)		
No	3 (3.0)	25 (4.3)		
Groups by severity (*n* = 617), *n* (%)			(8.132)	0.017
Mild	17 (18.7)	52 (9.9)		
Moderate	31 (34.1)	243 (46.2)		
Severe	43 (47.3)	231 (43.9)		
Age (*n* = 762), M±SD	68.12 ± 10.882	64.60 ± 12.816	3.650	<0.001
Diabetes history (year) (*n* = 675), *M* ± SD	13.04 ± 8.262	12.99 ± 8.092	0.058	0.954
Glycosylated hemoglobin (*n* = 402), M ± SD	9.75 ± 2.232	9.60 ± 2.409	0.294	0.769

The treatment of patients with diabetic foot may be closely related to the severity, and thus we analyze the differences of the indicators before and after reform in mild, moderate, and severe groups individually, which includes treatment and category of operations. Results show the disability score per 100 diabetic foot patients in the severe group decreased from 27.91 to 23.18. However, there is no statistical significance in the indicators of treatment and category of operations, which may be due to the limited sample size (Appendix 1).

### Effect Analysis

Among the 762 hospitalized patients with diabetic foot, there are 529 non-amputated patients and 233 amputated patients (177 phalange/metatarsal resections, 19 below-knee amputations, and 37 above-knee amputations). The number of non-amputated patients is significantly higher than that of amputated patients in the same period, and both show a substantial increase. The number of non-amputated patients has increased from 21 to 87, with an increase of 314.29% and an average annual increase of 15.27%; the number of amputated patients has increased from 8 to 48, with an increase of 500.00% and an average annual increase by 19.62%. The increase rate of amputated patients is higher than that of non-amputated patients, making the proportion of amputated patients increase from 27.59 to 35.56%, an increase of 7.97 percentage points (as Table 4 shows).

**Table 4:** Comparison of indicators of diabetic patients with non-amputated and amputated patients from 2009 to 2019.

	**Year**	**2009**	**2010**	**2011**	**2012**	**2013**	**2014**	**2015**	**2016**	**2017**	**2018**	**2019**	**Rate of average annual increase (%)**
Discharged patients (time)	Non-amputated patients	21	28	22	36	11	19	67	79	85	74	87	15.27
	Fixed-base growth rate* (%)	–	33.33	4.76	71.43	−47.62	−9.52	219.05	276.19	304.76	252.38	314.29	
	Amputated patients	8	4	11	6	5	13	20	27	36	55	48	19.62
	Fixed-base growth rate (%)	–	−50.00	37.50	−25.00	−37.50	62.50	150.00	237.50	350.00	587.50	500.00	
Proportion of amputated patients (%)		27.59	12.50	33.33	14.29	31.25	40.63	22.99	25.47	29.75	42.64	35.56	
Average length of stay (day)	Non-amputated patients	25.48	17.89	21.00	19.75	41.55	15.74	17.06	13.99	14.76	13.81	10.44	−8.53
	Fixed-base growth rate (%)	–	−29.77	−17.57	−22.48	63.08	−38.22	−33.04	−45.10	−42.05	−45.79	−59.02	
	Amputated patients	36.38	25.75	35.09	39.17	44.80	23.15	28.80	28.48	26.44	26.64	28.29	−2.48
	Fixed-base growth rate (%)	–	−29.21	−3.53	7.67	23.16	−36.35	−20.82	−21.70	−27.30	−26.77	−22.22	
Average hospitalization cost (rmb: yuan)	Non-amputated patients	13,343.38	12,517.32	19,114.22	17,076.05	23,268.22	13,723.76	21,182.69	19,471.81	20,389.54	22,096.67	15,009.15	1.83
	Fixed-base growth rate (%)	–	−6.19	43.25	27.97	74.38	2.85	58.75	45.93	52.81	65.60	12.48	
	Amputated patients	20,973.75	18,580.40	34,418.42	31,577.87	50,915.01	26,526.56	36,158.96	39,877.33	44,197.26	41,017.71	54,088.29	9.94
	Fixed-base growth rate (%)	–	−11.41	64.10	50.56	142.76	26.48	72.40	90.13	110.73	95.57	157.89	

#### Quality and Safety: Amputation Rate and Disability

Among 233 amputated patients, there are only 19 patients with above-knee amputation, and the cases are zero in some years. Because of the small number, according to the time of diabetic foot treatment collaboration, the description and analysis of amputated patients with three categories are divided into two stages: 2009–2014 and 2015–2019.

From 2009 to 2014, the number of phalange/metatarsal resection, below-knee amputation, and above-knee amputation is 30, 3, and 14, respectively, and the corresponding amputation rate is 16.30, 1.63, and 7.61%. From 2015 to 2019, the number of amputation cases has increased to 147, 16, and 23, respectively, and the amputation rate has changed to 25.43, 2.77, and 3.98%. The rate of phalange/metatarsal resection and below-knee amputation has increased, while the rate of above-knee amputation decrease. This probably relates to timely and early-stage intervention after collaborative treatment of internal medicine and surgery, which controls the progression of the disease and significantly improves the intervention effect (as Table 5 shows).

**Table 5:** Changes of the amputation rate (frequency) in patients with diabetic foot from 2009 to 2014 and 2015 to 2019.

	**2009–2014**	**2015–2019**
Phalange/metatarsal resection	16.30% (30/184)	25.43% (147/578)
Below-knee amputation	1.63% (3/184)	2.77% (16/578)
Above-knee amputation	7.61% (14/184)	3.98% (23/578)

Amputation of the diabetic foot leads to cause physical disability of the patient. On the January 14, 2011, General Administration of Quality Supervision, Inspection and Quarantine of the People's Republic of China and Standardization Administration jointly issued the “Classification and grading criteria of disability” (GB/T26341-2010), which have identified the lack of double thighs as Class II physical disabilities, the lack of a single thigh or double calves as Class III physical disabilities, the lack of a single calf or above the tarsometatarsal joint as Class IV physical disabilities.

Because of the incomplete electronization, it is unable to determine whether the amputation is a single thigh or double thighs in 8 cases and a single calf or double calves in 3 cases. In this study, statistical analysis is based on the single thigh and single calf amputation. From 2009 to 2014, a total of 10 cases of thigh amputation/disarticulation, 4 cases of knee joint disarticulation, and 3 cases of calf amputation/disarticulation were performed in Luhe Hospital. The disability score per 100 diabetic foot patients is 16.85. From 2015 to 2019, the hospital carried out 23 cases of thigh amputation/disarticulation, 8 cases of calf amputation/disarticulation, 2 cases of ankle joint disarticulation, and 6 cases of foot disarticulation. (The medical records did not indicate whether the foot disarticulation is above the tarsometatarsal joint. If foot disarticulation is above the tarsometatarsal joint, it is Class IV physical disabilities. Unless it does not reach the disability standard. In this study, it is considered that foot disarticulation is above the tarsometatarsal joint.) The disability score per 100 diabetic foot patients is 10.73 (If foot disarticulation is identified as below the tarsometatarsal joint, the disability score per 100 diabetic foot patients is 9.69), which is significantly lower than that of 2009–2014 (as Table 6 shows).

**Table 6:** Changes of the disability in patients with diabetic foot from 2009 to 2014 and 2015 to 2019.

**Disability classification**	**Category**	**2009–2014**	**2015–2019**
Class III physical disabilities	Thigh amputation/disarticulation (cases)	10	23
	Knee joint disarticulation (cases)	4	0
Class IV physical disabilities	Leg amputation/disarticulation (cases)	3	8
	Ankle joint disarticulation (cases)	0	2
	Foot disarticulation (cases)	0	6
Total number of diabetic foot patients in the same period		184	578
Disability score per 100 diabetic foot patients		16.85	10.73

[Disability Score: Class III Physical Disabilities is set to 2 points, Class IV Physical Disabilities is set to 1 point. Disability Score per 100 Diabetic Foot Patients = (the number of Class III Physical Disabilities ^*^ 2 + the number of Class IV Physical Disabilities ^*^ 1)/Total Number of Diabetic Foot Patients in the Same Period ^*^ 100].

#### Efficiency

From 2009 to 2019, the average length of stay of non-amputated patients is consistently lower than that of amputated patients in the same period. And both show a fluctuating downward trend. The average length of stay of non-amputated patients decreased from 25.48 to 10.44 days, with a decrease of 59.02% and an average annual decrease of 8.53%; the average length of stay of amputated patients decreased from 36.38 to 28.29 days, with a decrease of 22.22% and an average annual decrease of 2.48%, which is less than that of non-amputated patients (as Table 4 shows).

For amputated patients, the average length of stay of phalange/metatarsal resection patients decreases from 31.03 to 25.87 days, with a decrease of 16.64%; the average length of stay of above-knee amputation patients decreases from 37.71 to 30.70 days, with a decrease of 18.61%. There is no significant change in mean operation time for phalange/metatarsal resection patients, while there is an increase of 47.23% for above-knee amputation patients. The average length of post-operative stay decreased by 8.40% in phalange/metatarsal resection patients and 22.45% from 25.29 to 19.61 days in above-knee amputation patients (as Table 7 shows).

**Table 7:** Comparison of indicators for phalange/metatarsal resection, below-knee amputation and above-knee amputation patients during 2009–2014 and 2015–2019.

	**2009–2014**	**2015–2019**	**Growth rate (%)**
**Discharged patients (time)**			
Phalange/metatarsal resection	30	147	390.00
Below-knee amputation	3	16	433.33
Above-knee amputation	14	23	64.29
**Average length of stay (day)**			
Phalange/metatarsal resection	31.03	25.87	−16.64
Below-knee amputation	27.00	38.19	41.44
Above-knee amputation	37.71	30.70	−18.61
**Mean operation time (minute)**			
Phalange/metatarsal resection	53.33	52.45	−1.65
Below-knee amputation	…	92.86	-
Above-knee amputation	70.00	103.06	47.23
**Average length of post-operative stay (day)**			
Phalange/metatarsal resection	23.07	21.13	−8.40
Below-knee amputation	13.33	30.06	125.45
Above-knee amputation	25.29	19.61	−22.45
**Average hospitalization cost (rmb: yuan)**			
Phalange/metatarsal resection	25,772.77	38,065.81	47.69
Below-knee amputation	16,863.53	85,720.09	408.32
Above-knee amputation	41,844.87	55,477.62	32.58

#### Cost

From 2009 to 2019, the average hospitalization cost of non-amputated patients was consistently lower than that of amputated patients in the same period. From the growth trend, both present fluctuating growth. The average hospitalization cost of non-amputated patients increases by 1.83% annually, while the increase in amputated patients is more obvious. The average hospitalization cost of amputated patients increased from 20,973.7 to 54,088.29 yuan, with an increase of 157.89% and an average annual increase of 9.94% (as Table 4 shows).

For amputated patients, the average hospitalization cost of phalange/metatarsal resection patients and above-knee amputation patients increased by 47.70 and 32.58%, respectively (as Table 7 shows).

Note: Due to the small number of below-knee amputation patients, individual patients have a great influence on the indicators. In the text part, we do not compare the four indicators of patients with below-knee amputation: average length of stay, mean operation time, average length of post-operative stay, and average hospitalization cost.

## Discussion

Organizational innovations tend to follow a cycle of broad acceptance followed by widespread disenchantment, often with little or no evaluation of their effectiveness (14). And this research provides an initial empirical exploration of the effectiveness of organizational reform. The number of hospitalized patients with diabetic foot has increased significantly after the reform, indicating that patients' tendency to choose Luhe Hospital and patients' trust has improved. On the sight of quality, the disease progression is controlled more timely and effectively with the combined intervention of physician and surgeon, which is also proved by a significant decrease in the rate of above-knee amputation, the occurrence of Class III physical disabilities, and the disability score per 100 diabetic foot patients (Compared with 2009–2014, there was a significant decrease in the disability score per 100 diabetic foot patients in 2015–2019, regardless of whether grouped by severity or not). In terms of efficiency, more patients with surgical indications have received timely treatment, and the average length of stay is shortened significantly. Generally, the early intervention and treatment implemented effectively have improved the life quality of patients with diabetic foot. According to international standards, the diabetic foot could only be successfully treated by MDT, which provides more comprehensive and integrated care than one single specialist.

Amputation is an undesirable outcome for diabetic foot patients, and the quality of life will be significantly reduced (15). A severe amputation may result in an inability to work or even permanent dependence on assistance (16). Therefore, the prevention of major amputation is one of the main goals of diabetic foot treatment (17). In order to better evaluate the living ability of patients after surgery, we have created a new measurable indicator: disability score per 100 diabetic foot patients. In this study, the reform significantly reduce the incidence of Class III physical disabilities, and previous studies show MDT is the key to reducing diabetic foot amputation (18). However, the rate of phalange/metatarsal resection and below-knee amputation have increased, and the possible reason is that patients tended to suffer ulcers and/or gangrenes when hospitalized. It's often impossible to avoid minor amputations (15). In addition, patients with damage caused by long-term hyperglycemia usually need treatment for multiple organs. And MDT mainly involves endocrinology, vascular surgery, neurology, orthopedics, burn surgery, infection surgery, general surgery, and medical iconography (19). Early intervention of MDT is conducive to reducing the degree of disability (20). Therefore, it is necessary to establish diabetic foot MDT for early intervention.

Diabetic foot is the most common cause of hospitalization for diabetes (21). Compared with the traditional model, the reform is helpful to shorten the average length of stay. In this study, with the exception of below-knee amputation, patients have a significantly shorter average length of stay after the reform, which is consistent with the results of previous studies (22). Patients do not have to run around because of comprehensive and accurate diagnosis and treatment of the reform (23). The risk factors for major amputation are pre-operative and post-operative blood glucose regulation. Collaboration between vascular surgeons and endocrinologists brings a win-win proposition. As a result, the average post-operative stay of patients with phalange/metatarsal resection and above-knee amputation is also significantly shortened under principles of pre-operative preparation, intraoperative cooperation, and post-operative comprehensive overall nursing interventions.

“Guidelines on multidisciplinary approaches for the prevention and management of diabetic foot disease (2020 edition)” indicts that the medical cost of diabetes treatment in China will increase from the current $4.9 billion to over $7.4 billion in 2030. Based on the assumption that diabetic foot accounts for 20% of the total medical costs associated with diabetes, this would impose a heavy economic burden on society (24). Research has shown that managing diabetic foot in MDT can help reduce the economic burden of the disease (24). However, these other studies are conducted in high-income countries, and it is difficult to generalize the results to other countries because the health resources and expertise of managing the disease differ substantially in various settings worldwide, and economic outcomes may vary among various healthcare systems (25). In this study, the average hospitalization cost increases, whether the patient underwent surgery or not. Given that the cost of hospitalization also included the manpower and material resources of MDT, the increasing expense is reasonable. Another assumption is that reimbursement in China was based on DRGs payment. Different patients with the same disease may be divided into different groups for different main diagnoses, and then the DRGs payments will directly differ in the cost. As a matter of fact, our research is not sufficient, but the previous research (9) proves that MDT can reduce the life-cycle economic burden of diabetic foot patients.

Above all, the organ-system-based, disease-coring multidisciplinary health care project emphasizes a patient-centered approach, which is beneficial to better health outcomes. We have summarized the characteristics and advantages of the organizational reform, mainly including two contents, in the hope of being a reference and inspiration to other hospitals in the future.

- Luhe Hospital rearranges the spatial design of inpatient and outpatient departments to provide one-stop service. Patients are able to complete most of the examination, diagnosis and treatment procedures in the “Clinic District,” avoiding wasting time among different floors and buildings in the hospital. The spatial design rearrangement assures the effective and efficient execution of the care process, considering the minimization of patients move, efficiency lose of staff, and relocation cost.
- Compared to the traditional MDT, more combined cooperation is required in the reform, where multidisciplinary physicians share responsibilities, achievements, risks, and benefits. Each patient will receive personalized treatment. The clinical pathways are more standardized, and the multidisciplinary intervention is more precise, providing throughout MDT health care rather than an occasional one.

### Limitation

The study is retrospective. Compared with the prospective study, there are limitations in the selection of indicators for effective evaluation. Only available indicators are included.

## Conclusion

This study is designed to investigate a typical organizational reform in China and analyze its practical effects. The new measurable indicator called disability score per 100 diabetic foot patients has been verified to evaluate the living ability of patients after surgery. The diabetic foot care is complex. Appropriate and timely care requires multidisciplinary collaboration. The reform effectively shortens the length of hospital stay, reduces complications, and decreases the disability rate and disability score. Therefore, the organ-system-based, disease-coring multidisciplinary health care project could be promoted to more intersecting diseases. Importantly, it provides a new method for hospital construction for better service.

## Data Availability Statement

The data analyzed in this study is subject to the following licenses/restrictions: The dataset generated and analyzed during the current study are not publicly available due to the privacy protection of patient information. Requests to access these datasets should be directed to Mengchi Hou, mengchih@ccmu.edu.cn.

## Author Contributions

MH and XG conceived and designed the study, performed the statistical analysis, and wrote the paper. RG and ZJ obtained funding, contributed to the interpretation of the results, critical revision of the manuscript for important intellectual content, and approved the final version of the manuscript. MH, XG, WC, JD, FZ, ZJ, and RG collected the data. Moreover, all authors have approved the final manuscript for submission.

## Funding

This work was supported by grants from Capital's Funds for Health Improvement and Research (2018-2-7081) and National Natural Science Foundation of China (72174131).

## Conflict of Interest

The authors declare that the research was conducted in the absence of any commercial or financial relationships that could be construed as a potential conflict of interest.

## Publisher's Note

All claims expressed in this article are solely those of the authors and do not necessarily represent those of their affiliated organizations, or those of the publisher, the editors and the reviewers. Any product that may be evaluated in this article, or claim that may be made by its manufacturer, is not guaranteed or endorsed by the publisher.

## Abbreviations

MDT: The multidisciplinary team.
