# Supplementary material for: Will Multidisciplinary Collaboration Reduce the Disability Rate of Diabetic Foot (2009–2019)?—A Study Based on the Perspective of Organizational Reform
Source: Front Public Health. 2021 Oct 8;9:760440. doi: 10.3389/fpubh.2021.760440 (PMC8531470; doi:10.3389/fpubh.2021.760440)
Supplement: Supplementary file 1 [file Data_Sheet_1.PDF]

Appendix 1

Table 1 Comparison of indicators of diabetic patients in mild, moderate and severe groups from 2009-2014 and 2015-2019

	Mild, n=69				Moderate, n=272				Severe, n=276			
	2009-2014	2015-2019	χ^2	*P* value	2009-2014	2015-2019	χ^2	*P* value	2009-2014	2015-2019	χ^2	*P* value
Treatment, n (%)			-	-			＜0.001	＞0.999			2.966	0.085
Non-amputated	17 (100.0)	52 (100.0)			26 (83.9)	204 (84.6)			24 (55.8)	97 (41.6)		
Amputated	0 (0.0)	0 (0.0)			5 (16.1)	37 (15.4)			19 (44.2)	136 (58.4)		
Category of operations, n (%)			-	-			-	-			4.883	0.087
Phalange/Metatarsal Resection	0 (0.0)	0 (0.0)			5 (100.0)	37 (100.0)			13 (68.4)	102 (75.0)		
Below-knee Amputation	0 (0.0)	0 (0.0)			0 (0.0)	0 (0.0)			0 (0.0)	14 (10.3)		
Above-knee Amputation	0 (0.0)	0 (0.0)			0 (0.0)	0 (0.0)			6 (31.6)	20 (14.7)		
Disability Score per 100 Diabetic Foot Patients*	-	-			-	-			27.91	23.18		

*Disability Score: According to the "Classification and grading criteria of disability" (GB/T26341-2010) jointly issued by General Administration of Quality Supervision, Inspection and Quarantine of the People's Republic of China and Standardization Administration on January 14, 2011, we identified above-knee amputation as Class III physical disabilities, and below-knee amputation as Class IV physical disabilities. Class III Physical Disabilities is set to 2 points, and Class IV Physical Disabilities is set to 1 points. Disability Score per 100 Diabetic Foot Patients = (the number of Class III Physical Disabilities*2 + the number of Class IV Physical Disabilities*1) / Total Number of Diabetic Foot Patients in the Same Period*100).
